# Postmortem CT and autopsy findings in an elevator-related death: a case report

**DOI:** 10.1007/s12024-024-00896-3

**Published:** 2024-09-20

**Authors:** Giovanni Aulino, Michele Rega, Vittoria Rossi, Massimo Zedda, Antonio Oliva

**Affiliations:** Department of Health Surveillance and Bioethics, Section of Legal Medicine, Università Cattolica del Sacro Cuore, Fondazione Policlinico Universitario A. Gemelli IRCCS, Largo Francesco Vito, 1, Rome, 00168 Italy

**Keywords:** Virtopsy, Forensic radiology, Elevator accident, PMCT, Fracture

## Abstract

Elevator-related fatalities and injuries are rarely discussed. Falls have been identified as the first cause of mortality in the majority of these accidents. Evidence suggests that many elevator accidents may be attributed to inadequate equipment maintenance or malfunctions of the devices. This study examines a case involving an elevator maintenance worker found within an elevator shaft, using postmortem computed tomography (PMCT) along with a full autopsy. The autopsy revealed that the cause of death was severe polytrauma resulting from dragging, compression, and crushing mechanisms, which resulted in a dislocated skull and multiple thoraco-abdominal injuries, including exposed organs and viscera. Detailed examination identified a cranio-encephalic crush, leading to a significant alteration in the physiognomy of the facial structures. Additionally, PMCT revealed complex spinal fractures, such as a Jefferson fracture and a complete Chance fracture at the D6 vertebra, accompanied by spinal deviation proximal to the fracture site. Autopsy findings corroborated these PMCT results. A multidisciplinary approach, including PMCT, is proposed as a strategic method for the comprehensive reconstruction of such accidents, facilitating the collection of extensive data.

## Introduction

Elevator-related fatalities and injuries are infrequently addressed in literature. Between 1992 and 2009, there were 443 reported fatalities associated with elevators in the United States [1]. Conversely, injuries attributed to elevators are believed to occur more frequently within the United States, impacting both children and the elderly [2, 3]. In cases involving fall-related trauma, PMCT has facilitated a more accurate reconstruction of the accident and provided a detailed characterization of the injuries sustained, as corroborated by subsequent autopsy examinations [4].

In our institution, PMCT is utilized in specific forensic cases, including those involving extensive carbonization, gunshot wounds, and drowning. This technique was applied to assess a workplace accident involving an elevator, in which the victim was found motionless within the elevator shaft [5–9]. The injuries sustained from the precipitating event were significantly overshadowed by the presence of severe polytrauma, which led to cranio-encephalic displacement and multiple thoracic and abdominal injuries, including exposed organs and viscera. Consequently, this study aims to provide a comprehensive description of the fractures sustained, integrating evidence from the accident scene with radiological and autopsy findings.

## Case report

### Case presentation

A 35-year-old elevator maintenance worker was discovered in a prone position at the base of the elevator shaft where he had conducted maintenance the preceding day. He had been reported missing since the previous evening. The individual was found wearing a bloodstained shirt and trousers that were partially lowered to just above the knees, along with work shoes, of which only the left shoe was worn. Additionally, longitudinal streaks of blood were noted on the walls of the upper compartment at the scene.

### External examination

**Fig. 1:**
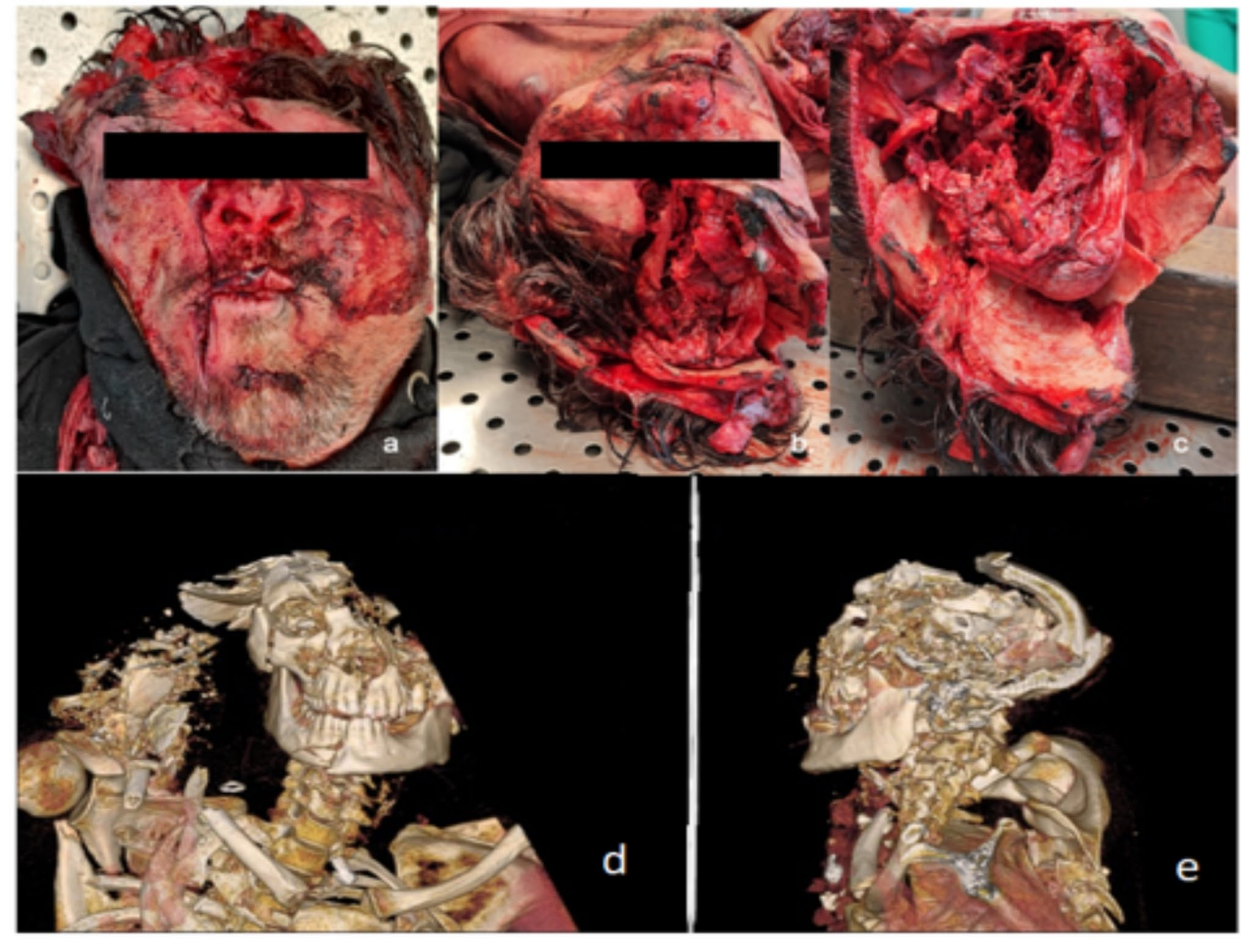
External examination and PMCT findings of the craniofacial region. **a, b, c**: Cranio-encephalic injury characterized by significant loss of facial structure, including ocular rupture and destruction of the bony components of the cranial vault. **d, e**: PMCT 3D-rendered images depicting the bone structure of the craniofacial area

Upon examination of the head, a cranio-encephalic crush was identified, characterized by significant disruption of the facial structure, including disrupted eyes and destruction of the bony elements of the cranial vault. Comminuted fragments of cerebral parenchyma were also present (Fig. 1a-c).

**Fig. 2:**
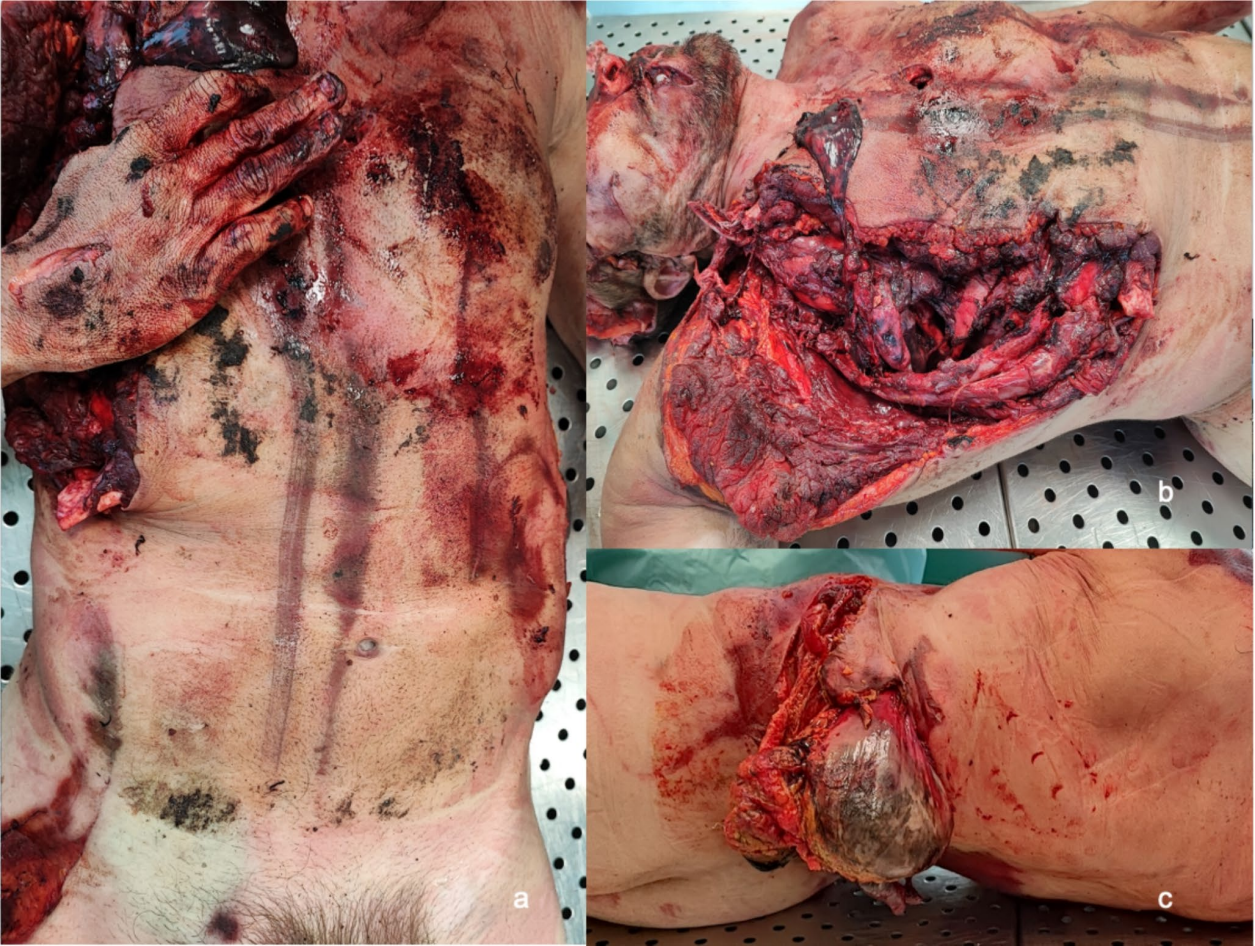
External examination findings of the thoraco-abdominal region. **a:** Two parallel ecchymotic and excoriated bands measuring 1 cm in thickness, located in the mid-sternal region and adjacent laterally. **b:** A lacerated-contused wound with exposure of underlying musculoskeletal tissue and homolateral lung parenchyma in the right thoracic region. **c:** A lacerated-contused wound with a longer transverse axis, exposing abdominal viscera in the left flank

Two parallel ecchymotic and excoriated bands were observed: one in the mid-sternal region and another laterally adjacent, each approximately 1 cm thick and diffusely over the anterior thorax and abdomen. Additionally, lacerated and contused wounds affecting the anterior trunk were observed (Fig. 2a).

**Fig. 3:**
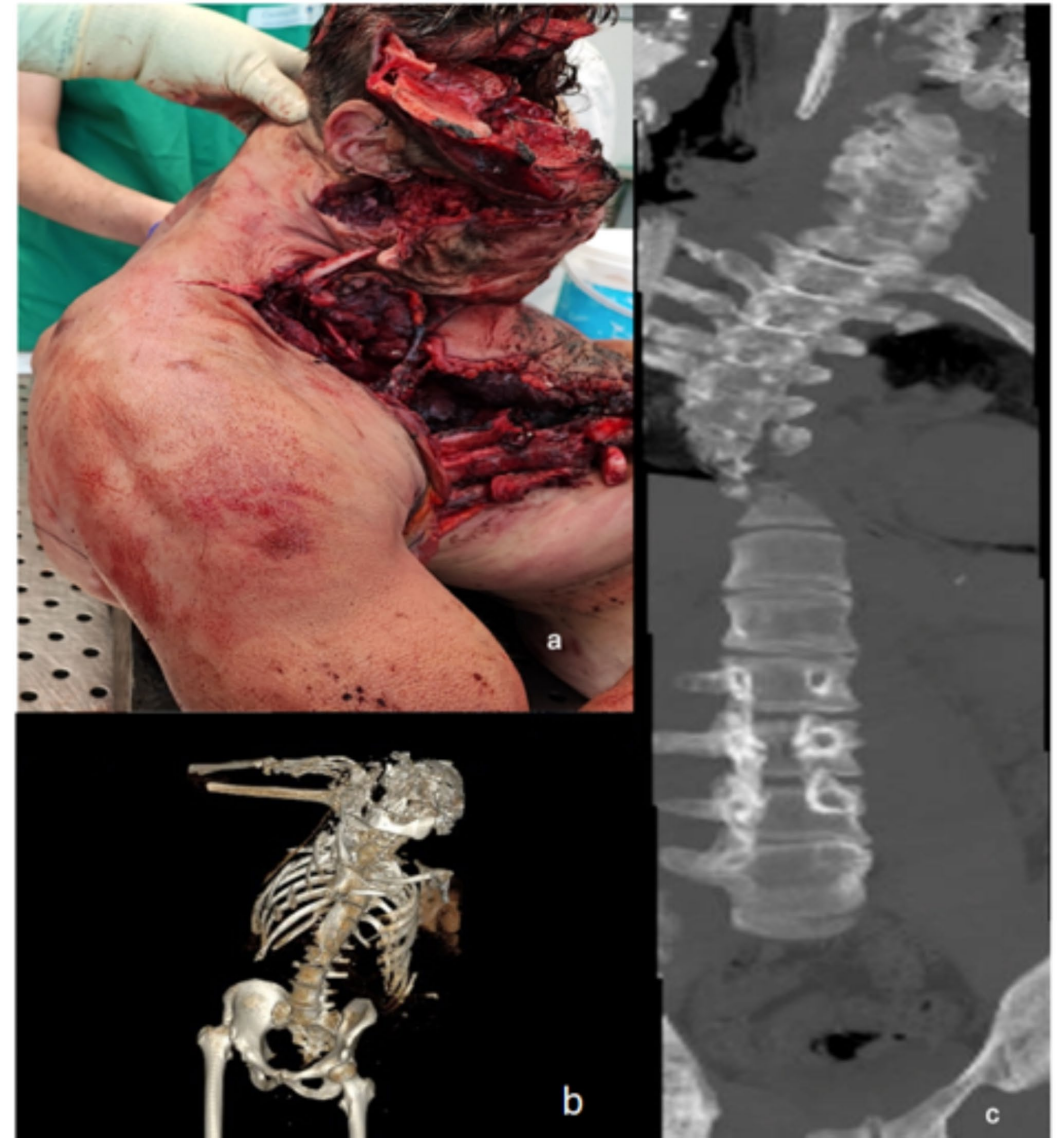
External examination and PMCT findings of the spinal column. **a:** Full-thickness fracture of the thoracic spine demonstrated. **b:** PMCT 3D-rendered image of the spinal column’s bone tissue. **c:** Frontal view of the spine as observed on the PMCT scan

In the right thoracic region, a lacerated contusion was identified, associated with multiple dislodged rib fractures and leakage of lung parenchyma. A similarly sized injury was found on the left flank, with exposure of the abdominal viscera (Fig. 2b, c). Finally, a full-thickness fracture of the thoracic vertebra was documented (Fig. 3a).

### Post-mortem CT

Prior to the autopsy, and following the external examination of the decedent, PMCT was conducted using a Somatom Sensation 16 CT scanner (Siemens^®^, Munich, Germany). The examination settings included 140 kVp, 160 mAs, 24-mm feed/rotation, 1-mm slice collimation, 1-mm slice width, and reconstruction kernels of 10, 30, 40, 70, and 80.

Subsequent to data processing, axial, coronal, and sagittal two-dimensional (2D) reconstructions, along with a three-dimensional (3D) volume rendering (VR) and shaded surface display (SSD), were performed. The resultant images were reviewed by a board-certified radiologist with over 10 years of experience in forensic imaging. The findings from the external examination were communicated to the radiologist prior to the review.

PMCT confirmed fractures in the craniofacial region (Fig. 1d, e) and revealed complex fractures of the spine, including a Jefferson fracture and a complete Chance fracture at the D6 level, with spinal deviation proximal to the fracture (Fig. 3b, c). Numerous fractures were also identified in all four limbs as well as in the thoracic cage. Additionally, a diaphragmatic rupture and mediastinal dislocation were noted.

### Autopsy findings

All radiological findings were corroborated by the forensic autopsy. The macroscopic examination revealed multiple lacerations of the thoracic and abdominal viscera. On sectioning, pronounced pallor of the organs was observed. Moreover, no significant findings identified that would suggest the presence of underlying pathologies capable of contributing to the determination of death.

## Discussion

Elevator accidents, while infrequent, can lead to fatalities and severe injuries [10]. Despite being one of the safest forms of transportation, the high volume of elevator traffic can contribute to serious incidents [2]. According to McCann, 20.5% of elevator passengers were not engaged in work at the time of their accidents, while 20.1% were performing work-related duties, such as clerical, stock handling, and janitorial tasks. Notably, the remaining cases (59.4%) involved construction workers who were in or near elevator shafts [1].

Reconstructing elevator-related accidents is crucial for both safety improvement and legal accountability [4]. The majority of these accidents can often be attributed to inadequate maintenance or malfunctioning equipment [6]. The nature of injuries sustained in elevator incidents varies according to the specific circumstances surrounding each case. Prahlow et al. highlighted that falls from heights were the leading cause of elevator-related fatalities, followed closely by severe asphyxia, crushing injuries, and pressure-related injuries, which placed third [11].

In this case, the cause of death was determined to be severe polytrauma resulting from dragging, compression, and crushing injuries that led to a dislocated skull and multiple thoraco-abdominal injuries, exposing internal organs and viscera. The most plausible scenario suggests that the victim experienced compression between the elevator shaft wall and the elevator, followed by a fall that resulted in further crushing as the elevator descended.

Evidence supporting this hypothesis includes blood found on the wall adjacent to the elevator, facial lacerations, longitudinal bruising on the anterior chest, and multiple fractures identified through PMCT. It has demonstrated not only exceptional sensitivity and specificity in recognizing and classifying various types of fractures, but it also allows for the identification of injuries to soft tissues and organs [12–16].

A systematic review of 15 studies comparing PMCT and autopsy findings in cases of traumatic death indicated an agreement rate ranging from 50 to 100% in determining the cause of death, with enhanced concordance noted specifically in gunshot-related fatalities [17]. Additionally, a subsequent large-scale study revealed an almost perfect correlation between PMCT and autopsy results in the detection of craniofacial injuries and gunshot-related deaths [18]. Thus, PMCT was instrumental in characterizing fractures that would have been challenging to analyze during a standard autopsy, thereby providing critical insights into the dynamics of the accident [19]. Indeed, PMCT has enabled the description and reconstruction of fractures in the craniofacial region and has revealed complex spinal fractures, including a Jefferson fracture and a complete Chance fracture at the D6 level, along with spinal deviation proximal to the fracture.

The autopsy further confirmed the absence of any pre-existing pathological conditions that could have contributed to the victim’s injuries. To maximize data collection, a multidisciplinary approach incorporating PMCT is essential [20]. Nevertheless, the autopsy remains a key component in establishing the cause of death and ruling out any underlying health issues that may have played a role in the injuries sustained.

In conclusion, the circumstances of the accident and the height of the fall significantly influence the severity of injuries and the likelihood of a fatal outcome in elevator incidents. Precise reconstruction of these accidents is essential for forensic investigations, as it aids in understanding the dynamics involved, especially in industrial contexts. Additionally, PMCT has proven to be a valuable, rapid, and non-invasive tool for the documentation and reconstruction of traumatic injuries, enhancing the overall forensic analysis.

## Data Availability

My manuscript has associated data in a data repository.

## Declarations

### Article classification

10: Forensics.

10.010: Pathology.

10.190: Autopsy.
